# Susceptibility of SARS-CoV-2 Omicron Variants to Therapeutic Monoclonal Antibodies: Systematic Review and Meta-analysis

**DOI:** 10.1128/spectrum.00926-22

**Published:** 2022-06-14

**Authors:** Kaiming Tao, Philip L. Tzou, Sergei L. Kosakovsky Pond, John P. A. Ioannidis, Robert W. Shafer

**Affiliations:** a Division of Infectious Diseases, Department of Medicine, Stanford University, Stanford, California, USA; b Institute for Genomics and Evolutionary Medicine, Temple University, Philadelphia, Pennsylvania, USA; c Departments of Medicine and of Epidemiology and Population Health and Meta-Research Innovation Center at Stanford (METRICS), Stanford University, Stanford, California, USA; Kumamoto University

**Keywords:** SARS-CoV-2, Omicron variant, monoclonal antibody, neutralization, spike protein, COVID-19, antiviral therapy, multidrug resistance

## Abstract

SARS-CoV-2 Omicron variants contain many mutations in its spike receptor-binding domain, the target of all authorized monoclonal antibodies (MAbs). Determining the extent to which Omicron variants reduced MAb susceptibility is critical to preventing and treating COVID-19. We systematically reviewed PubMed and three preprint servers, last updated 11 April 2022, for the *in vitro* activity of authorized MAbs against the Omicron variants. Fifty-one studies were eligible, including 50 containing Omicron BA.1 susceptibility data and 17 containing Omicron BA.2 susceptibility data. The first two authorized MAb combinations, bamlanivimab/etesevimab and casirivimab/imdevimab, were largely inactive against the Omicron BA.1 and BA.2 variants. In 34 studies, sotrovimab displayed a median 4.0-fold (interquartile range [IQR]: 2.6 to 6.9) reduction in activity against Omicron BA.1, and in 12 studies, it displayed a median 17-fold (IQR: 13 to 30) reduction in activity against Omicron BA.2. In 15 studies, the combination cilgavimab/tixagevimab displayed a median 86-fold (IQR: 27 to 151) reduction in activity against Omicron BA.1, and in six studies, it displayed a median 5.4-fold (IQR: 3.7 to 6.9) reduction in activity against Omicron BA.2. In eight studies against Omicron BA.1 and six studies against Omicron BA.2, bebtelovimab displayed no reduction in activity. Disparate results between assays were common. For authorized MAbs, 51/268 (19.0%) results for wild-type control variants and 78/348 (22.4%) results for Omicron BA.1 and BA.2 variants were more than 4-fold below or 4-fold above the median result for that MAb. Highly disparate results between published assays indicate a need for improved MAb susceptibility test standardization or interassay calibration.

**IMPORTANCE** Monoclonal antibodies (MAbs) targeting the SARS-CoV-2 spike protein are among the most effective measures for preventing and treating COVID-19. However, SARS-CoV-2 Omicron variants contain many mutations in their spike receptor-binding domains, the target of all authorized MAbs. Therefore, determining the extent to which Omicron variants reduced MAb susceptibility is critical to preventing and treating COVID-19. We identified 51 studies that reported the *in vitro* susceptibility of the two main Omicron variants BA.1 and BA.2 to therapeutic MAbs in advanced clinical development, including eight authorized individual MAbs and three authorized MAb combinations. We estimated the degree to which different MAbs displayed reduced activity against Omicron variants. The marked loss of activity of many MAbs against Omicron variants underscores the importance of developing MAbs that target conserved regions of spike. Highly disparate results between assays indicate the need for improved MAb susceptibility test standardization.

## INTRODUCTION

Neutralizing antibodies (Abs) block the entry of virus into host cells and may also recruit host effector pathways to destroy virus-infected cells. Most SARS-CoV-2-neutralizing Abs identified in persons recovering from COVID-19 bind the surface-exposed spike receptor-binding domain (RBD) or N-terminal domain (NTD). The RBD is the main target of human neutralizing Abs and the sole target of those monoclonal antibodies (MAbs) that either have received emergency use authorization by the U.S. Food and Drug Administration or are in advanced clinical development. The RBD, which encompasses residues 306 to 534, alternates between a closed/down position and an open/up position. When in the up position, it binds to the human ACE2 receptor. Approximately 20 RBD residues form contacts with the human ACE2 receptor (1). The region of the RBD that contains these residues encompasses residues 438 to 506 and is called the receptor-binding motif, whereas the remainder of the RBD is called the RBD core.

Although no two SARS-CoV-2-neutralizing MAbs have identical epitopes, those binding the RBD have been grouped into several classes depending on the location of their binding residues and whether they can bind the RBD in its up and/or down position (2–4). According to the most used classification, class I and II MAbs bind to amino acids contained within the receptor-binding motif, while class III and IV MAbs bind solely or predominantly to the RBD core (3).

Five MAb preparations have been authorized by the U.S. FDA (5), two have been authorized in other countries, and 13 others are in phase II or III clinical trials (6). The combinations of bamlanivimab/etesevimab and casirivimab/imdevimab were authorized for outpatient treatment and postexposure prophylaxis in high-risk individuals. The combination cilgavimab/tixagevimab was authorized for preexposure prophylaxis in high-risk individuals. Sotrovimab and bebtelovimab were each authorized for the outpatient treatment of high-risk individuals.

The Omicron BA.1 variant contains 15 RBD mutations including G339D, S371L, S373P, S375F, K417N, N440K, G446S, S477N, T478K, E484A, Q493R, G496S, Q498R, N501Y, and Y505H. Mutations K417N, G446S, Q493R, G496S, Q498R, N501Y, and Y505H are located in the ACE2-binding site (1). The Omicron BA.2 variant contains three additional RBD mutations, T376A, D405N, and R408S, but does not contain G446S and G496S. Approximately 10% to 30% of Omicron BA.1 isolates also contain R346K (classified as BA.1.1). From previously published high-throughput studies in which all single RBD mutations were evaluated for their strength of binding to FDA-authorized MAbs, it was already known that that bamlanivimab/etesevimab (K417N, E484A, Q493R) and casirivimab/imdevimab (N440K, G446S, Q493R) would likely be inactive against the Omicron variant (7, 8). However, alone, none of the Omicron mutations were previously found to reduce susceptibility to cilgavimab, tixagevimab, sotrovimab, or bebtelovimab. Furthermore, because every combination of mutations cannot possibly be tested in advance, it was unclear what effect the mutations would have on these specific MAbs. We therefore systematically reviewed those studies that assessed the neutralizing activity of FDA-authorized MAbs against Omicron variants.

## RESULTS

### Search results.

**FIG 1:**
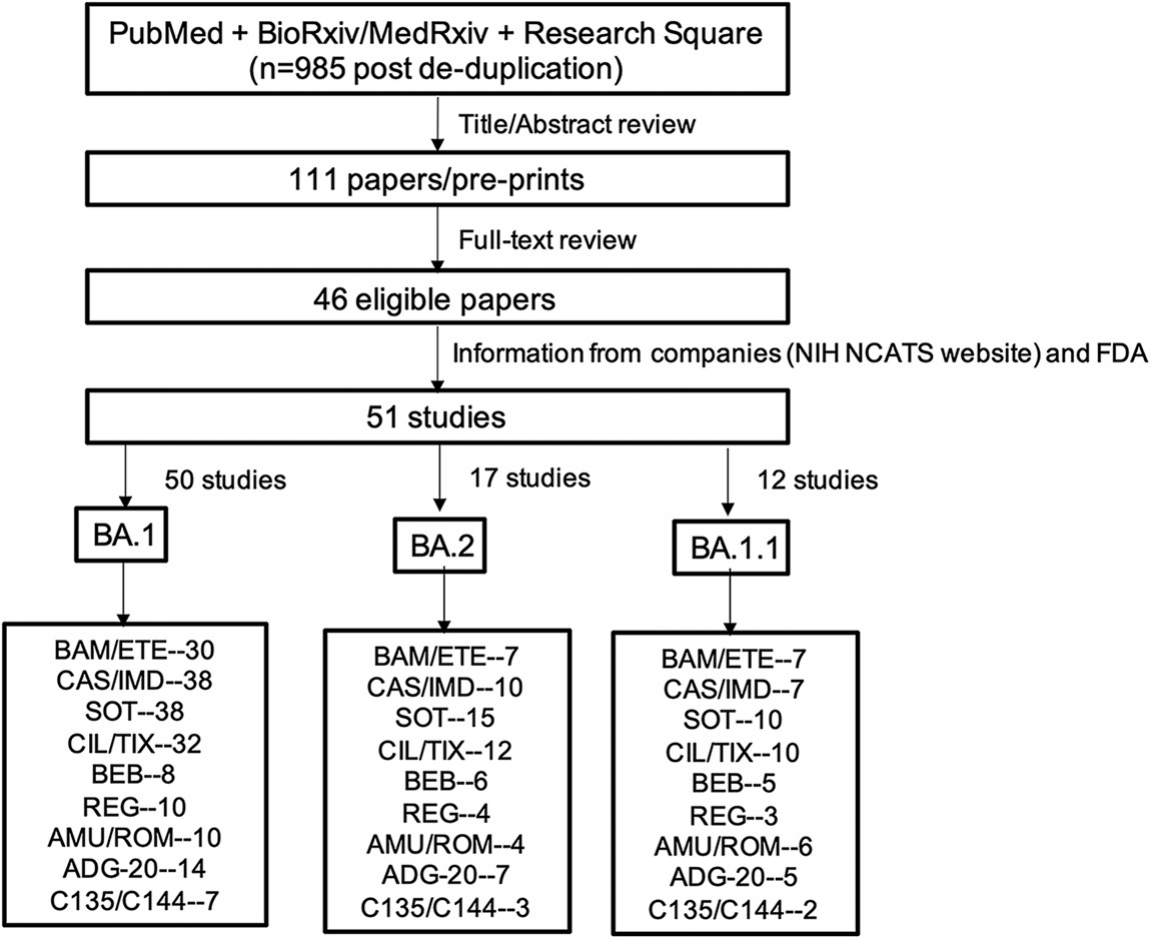
Flow chart of study selection process. Of 985 deduplicated studies identified through a search of PubMed and three preprint servers using the search string “SARS-CoV-2 AND Omicron AND (Neutralization OR Antibody OR Treatment),” 111 were read in their entirety following an initial review of titles and abstracts. Forty-six studies met our inclusion criteria in that they contained neutralizing susceptibility data for one or more FDA-authorized monoclonal antibodies (MAbs). Five additional data sets, including three FDA fact sheets and two data sets available on the NIH NCATs website, were also included. The number of studies containing susceptibility data for the Omicron BA.1 and BA.2 variants and the number of studies for each of the clinical-stage MAbs are shown. BAM, bamlanivimab; ETE, etesevimab; CAS, casirivimab; IMD, imdevimab; SOT, sotrovimab; CIL, cilgavimab; TIX, tixagevimab; REG, regdanvimab; ADI, adintrevimab; BEB, bebtelovimab; AMU, amubarvimab; ROM, romlusevimab. The presence of two MAbs separated by “/” indicates the combination was tested and/or that each individual MAb in the combination was also tested.

As of 11 April 2022, 46 studies met the search criteria (Fig. 1). Twenty-four of the studies were reported in a peer-reviewed publication (9–32); 22 were published as preprints (33–54). Three additional data sets were available from the FDA (55–57); two studies containing three additional data sets were available on the NIH National Center for Advancing Translational Sciences (NCATS) SARS-CoV-2 Variants and Therapeutics open data portal (58, 59). Neutralizing data for the Omicron BA.1, BA.2, and BA.1.1 variants were reported in 50, 17, and 12 studies, respectively (Fig. 1).

**FIG 2:**
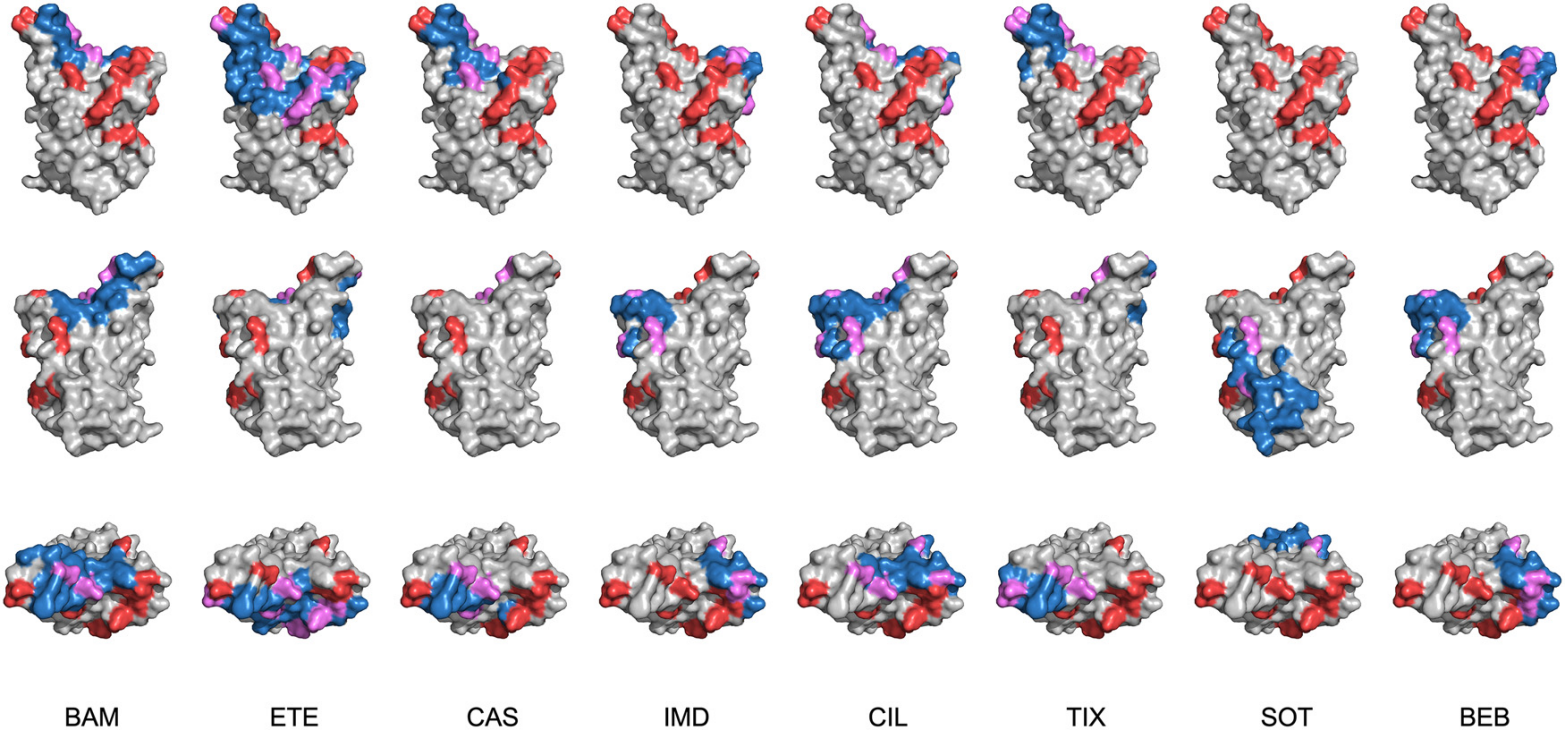
For each MAb, the top of the RBD and two side views are depicted using coordinates from PDB 6M0J. Positions mutated in Omicron BA.1, BA.1.1, and BA.2 are shown in red. The MAb epitope is shown in dark blue. Those positions at which Omicron mutations overlap the MAb epitope are shown in purple. The MAb epitopes for bamlanivimab (BAM), etesevimab (ETE), casirivimab (CAS), imdevimab (IMD), cilgavimab (CIL), tixagevimab (TIX), sotrovimab (SOT), and bebtelovimab (BEB) were determined from their PDB structures.

For the first two FDA-authorized MAb preparations, data for bamlanivimab, etesevimab, or the combination bamlanivimab/etesevimab were reported in 31 studies while data for casirivimab, imdevimab, or the combination casirivimab/imdevimab were reported in 39 studies. For the third and fourth FDA-authorized MAb preparations, data for sotrovimab were reported in 39 studies while data for cilgavimab, tixagevimab, or the combination cilgavimab/tixagevimab were reported in 33 studies (Fig. 1). Data for the most recently authorized MAb, bebtelovimab, were reported in 8 studies. The locations of each of the Omicron BA.1 and BA.2 RBD mutations and the epitopes of each of the authorized MAbs are displayed in Fig. 2.

### Neutralizing susceptibility assays.

Seven of the studies used two different assays to determine neutralizing activity; thus, overall, 58 assays were used by the 51 studies (11, 26, 39, 55–58). Sixteen of the studies used just authentic virus isolates, 29 studies used just pseudotyped virus isolates, and six studies used both authentic and pseudotyped viruses (Table 1). Of the 35 assays using pseudotyped viruses, 15 employed an HIV-1 backbone, 14 employed a vesicular stomatitis virus (VSV) backbone, one employed a murine leukemia virus (MLV) backbone, and one employed SARS-CoV-2 virus-like particles comprising the spike, nucleocapsid, membrane, and envelope structural proteins and a packaging signal-containing mRNA (47); for four studies the virus backbone was not indicated (Table 1).

**TABLE 1:** Descriptive overview of the neutralizing susceptibility assays and the MAbs undergoing testing

Author(s) (reference[s])	Virus type^*a*^	Cell line^*b*^	Inoculum^*c*^	Hours^*d*^	Control	Variant(s)	MAb(s)^*e*^
Aggarwal21 (33)	Infectious	Vero	10^4^	24	A.2.2	BA.1	BAM, CAS/IMD, SOT, CIL/TIX
Boschi22 (9)	Infectious	Vero	NA^*g*^	48	B.1	BA.1	BAM/ETE, CAS/IMD, CIL/TIX
Cameroni21 (11)	Infectious	Vero	NA	20	B.1	BA.1	SOT
Dejnirattisai22 (14)	Infectious	Vero	10^2^	NA	B	BA.1	BAM/ETE, CAS/IMD, SOT, CIL/TIX, ADI
Duty22 (39)	Infectious	Vero	10^2^	NA	A	BA.1	CIL/TIX, SOT
Fenwick22 (40)	Infectious	Vero	10^3^	48	Delta	BA.1, BA.2	CAS/IMD, CIL/TIX, SOT, ADI
Ma22 (43)	Infectious	Vero	10^2^	96	A	BA.1	BAM/ETE, CAS/IMD, SOT
Case22 (36)	Infectious	Vero-TMPRSS2	10^2^	60	B.1	BA.1, BA.1.1, BA.2	CIL/TIX, SOT
Ohashi22 (44)	Infectious	Vero-TMPRSS2	NA	24	A	BA.1, BA.2	CAS/IMD, SOT
Takashita22 and Takashita22b (30, 31)	Infectious	Vero-TMPRSS2	10^3^	24	A	BA.1, BA.2	BAM/ETE, CAS/IMD, SOT, CIL/TIX
Touret22 (25); Touret22b (48)	Infectious	Vero-TMPRSS2	NA	48	B.1	BA.1, BA.2	BAM/ETE, CAS/IMD, SOT, CIL/TIX, REG
VanBlargan22-1 (26)	Infectious	Vero-TMPRSS2	10^2^	70	B.1	BA.1	BAM/ETE, CAS/IMD, SOT, CIL/TIX, REG
VanBlargan22-2 (26)	Infectious	Vero-ACE2-TMPRSS2	10^2^	24	B.1	BA.1	BAM/ETE, CAS/IMD, SOT, CIL/TIX, REG
Meng22 (21)	Infectious	HOS-ACE2-TMPRSS2	10^2^	24	Delta	BA.1	CAS/IMD
Planas21 (22); Bruel22 (10)	Infectious	U2OS-ACE2	NA	20	Delta	BA.1, BA.2	BAM/ETE, CAS/IMD, SOT, CIL/TIX, REG, ADI
Wilhelm21 (51)	Infectious	NA	4 × 10^3^	48	B	BA.1	CAS/IMD
FDA21, FDA21b, and FDA22 (55–57)	Infectious	NA	NA	NA	NA	BA.1, BA.2, BA.1.1	CIL/TIX, SOT, BEB
Ai22 (34)	PV (VSV)	Vero	NA	24	B	BA.1, BA.1.1, BA.2	BAM/ETE, CAS/IMD, SOT, CIL/TIX, BEB, REG, AMU, ADI
Cameroni21 (11)	PV (VSV)	Vero	NA	20	B.1	BA.1, BA.1.1	BAM/ETE, CAS/IMD, SOT, CIL/TIX, REG
Cathcart22 (37)	PV (VSV)	Vero	2 × 10^3^	6	-	BA.1, BA.1.1, BA.2	SOT
Hoffmann22 (16); Schulz22 (23)	PV (VSV)	Vero	NA	16	B.1	BA.1	BAM/ETE, CAS/IMD, SOT, CIL/TIX
Rothenberger21 (46)	PV (VSV)	Vero	250	16	B	BA.1	BAM/ETE, CAS/IMD, SOT, CIL/TIX, AMU/ROM
Wang22 (28)	PV (VSV)	Vero	NA	24	B.1	BA.1	BAM/ETE, CAS/IMD, SOT, CIL/TIX, REG, ADI
Iketani22 and Liu21 (17, 19)	PV (VSV)	Vero	NA	10	B.1	BA.1, BA.1.1, BA.2	BAM/ETE, CAS/IMD, SOT, CIL/TIX, AMU/ROM, ADI, BEB
Duty22 (39)	PV (VSV)	293T-ACE2-TMPRSS2	NA	NA	B.1	BA.1, BA.1.1	CIL/TIX
Cao21, Cao22, and Cui22 (12, 13, 35)	PV (VSV)	Huh-7	10^3^	24	B.1	BA.1, BA.1.1, BA.2	BAM/ETE, CAS/IMD, SOT, CIL/TIX, BEB, ADI, AMU/ROM, C144
Wang22c (27)	PV (VSV)	Huh-7	NA	24	B	BA.1	ETE, IMD, TIX, SOT
Westendorf21 (50); FDA22 (56)	PV (VSV)	293T-ACE2/ACE2-TMPRSS2	NA	72	B.1	BA.1, BA.2	BAM/ETE, CAS/IMD, SOT, CIL/TIX, BEB, REG, ADI, C135/C144
FDA21, FDA21b, and FDA22 (55–57)	PV	293T-ACE2-TMPRSS2	NA	48	B	BA.1, BA.1.1, BA.2	CIL/TIX, SOT, BEB
NIH-NCATS21-2 (AstraZeneca) (Monogram) (58)	PV	293T-ACE2-TMPRSS2	1 × 10^4^–5 × 10^5^ RLU	72	B	BA.1	CIL/TIX
Lusvarghi22 (20)	PV (HIV)	293T-ACE2-TMPRSS2	1 × 10^5^–5 × 10^5^ RLU	48	B.1	BA.1	BAM/ETE, CAS/IMD, SOT, CIL/TIX, BEB, AMU/ROM, ADI, C144
Chen22 (38)	PV (HIV)	293T-ACE2-TMPRSS2	NA	72	A	BA.1	BAM/ETE, CAS/IMD
Zhou21 (29)^*f*^	PV (HIV)	293T-ACE2-TMPRSS2	NA	72	B.1	BA.1	BAM/ETE, CAS/IMD, SOT, CIL/TIX, BEB, REG, ADI, C135/C144
Gruell22 and Gruell22b (15, 41)	PV (HIV)	293T-ACE2	NA	48	B	BA.1, BA.1.1, BA.2	BAM/ETE, CAS/IMD, CIL/TIX, SOT, BEB, REG, ADI, AMU, C135/C144
Ju22 (18)	PV (HIV)	293T-ACE2	NA	48	B	BA.1	ETE, CAS/IMD, SOT, C144
Pelzek22 (45)	PV (HIV)	293T-ACE2	NA	60	B	BA.1	SOT
Sheward22 (24)	PV (HIV)	293T-ACE2	5 × 10^4^ RLU	48	B.1	BA.1	BAM/ETE, CAS/IMD, SOT
Tada22 and Zhou22 (32, 54)	PV (HIV)	293T-ACE2	2 × 10^3^	48	B.1	BA.1, BA.2	BAM/ETE, CAS/IMD, SOT, CIL/TIX
Wang22b (49)	PV (HIV)	293T-ACE2	NA	48	B	BA.1	IMD, SOT
Ikemura21 (42)	PV (HIV)	293T-ACE2	NA	48	B	BA.1	CAS/IMD, SOT
Yamasoba22 (52)	PV (HIV)	HOS-ACE2-TMPRSS2	2 × 10^4^ RLU	48	B.1	BA.1, BA.2	CAS/IMD, SOT
NIH-NCATS21 (Brii Biosciences) (59)	PV (HIV)	NA	NA	NA	B	BA.1	AMU/ROM
Yuan22 (53)	PV (MLV)	Vero	NA	120	B	BA.1	BAM/ETE, CAS/IMD, CIL/TIX, SOT, ADI, C144

a The reference is indicated by the first author’s surname followed by the year of publication. For authors that have more than one publication in the same year, a lower case letter has been added. For publications with more than one assay, a dash followed by a number has been added. PV, pseudotyped virus; HIV, human immunodeficiency virus; VSV, vesicular stomatitis virus; MLV, murine leukemia virus. A study using virus-like particles is not shown (47).

b Cell line followed by ACE2 and/or TMPRSS2 indicates cells modified to stably express these surface proteins.

c Studies using infectious viruses reported the inoculum as 50% tissue culture infectious doses (TCID_50_), focus-forming units (FFU), infectious units (IU), or multiplicity of infection (MOI). This column treats the TCID_50_, FFU, and IU similarly. MOI was used to calculate the inoculum if the number of cells per well was available. Studies using PVs inconsistently reported the virus inoculum, and when reported, it was reported as a TCID_50_ or as relative light units (RLU).

d The endpoint for the infectious virus assays was cytopathic effect usually augmented by immunostaining of virally infected cells, with the exception of two studies which used RNA yield (25, 48). PV assays measured RLU produced by luciferase-encoding reporter genes.

e BAM, bamlanivimab; ETE, etesevimab; CAS, casirivimab; IMD, imdevimab; SOT, sotrovimab; CIL, cilgavimab; TIX, tixagevimab; BEB, bebtelovimab; REG, regdanvimab; ADI, adintrevimab; AMU, amubarvimab; ROM, romlusevimab. The presence of two MAbs separated by “/” indicates the combination was tested and/or that each individual MAb in the combination was also tested. Not all MAbs were tested for activity against each of the Omicron variants. A dash indicates that the relevant data were not identified.

f 293T-ACE2 cells were used for TIX and 293T-ACE2-TMPRSS2 used for CIL. Several of the results in this study overlap results in Westendorf21 (50).

g NA, not available.

The spike mutations reported in each study were in nearly all cases identical to the prototype Omicron BA.1, BA.1.1, and BA.2 sequences. The exceptions included two studies in which the Omicron BA.1 sequence also contained A701V (9, 14) and three studies in which the pseudotyped virus spike contained Q493K rather than Q493R (32, 46, 54). Several studies explicitly reported using pseudotyped viruses containing spike cytoplasmic tail truncations (16, 23, 32, 36, 42, 54). This is a common practice used to increase the expression of spike proteins that may not have been consistently reported.

The most commonly used cell lines were Vero cells, Vero cells stably expressing TMPRSS2 or TMPRSS2 plus ACE2, and 293T cells stably expressing ACE2 or TMPRSS2 plus ACE2 (Table 1). Huh-7, HOS-ACE2-TMPRSS2, and U2OS-ACE2 cells were also used. Because Vero cells are intrinsically resistant to HIV-1 infection, they were not used in any of the assays using HIV-1 pseudotyped viruses. While most assays used ≥10,000 cells in 96-well plates, this information was not consistently reported. Similarly, the viral inoculum size was also inconsistently reported (Table 1).

For infectious virus assays, virus replication was assessed based on microscopic cytopathological effects usually accentuated with SARS-CoV-2 immunostaining (e.g., focus-forming or plaque-reduction assays). For pseudotyped virus assays, entry into cells was measured as relative light units as each virus construct contained a luciferase reporter gene. For most assays, the highest MAb concentrations were ≥10,000 ng/mL. Dose-response curves were included for 32 of the 51 studies.

### Omicron BA.1 variant neutralizing activity. (i) Bamlanivimab/etesevimab and casirivimab/imdevimab.

**FIG 3:**
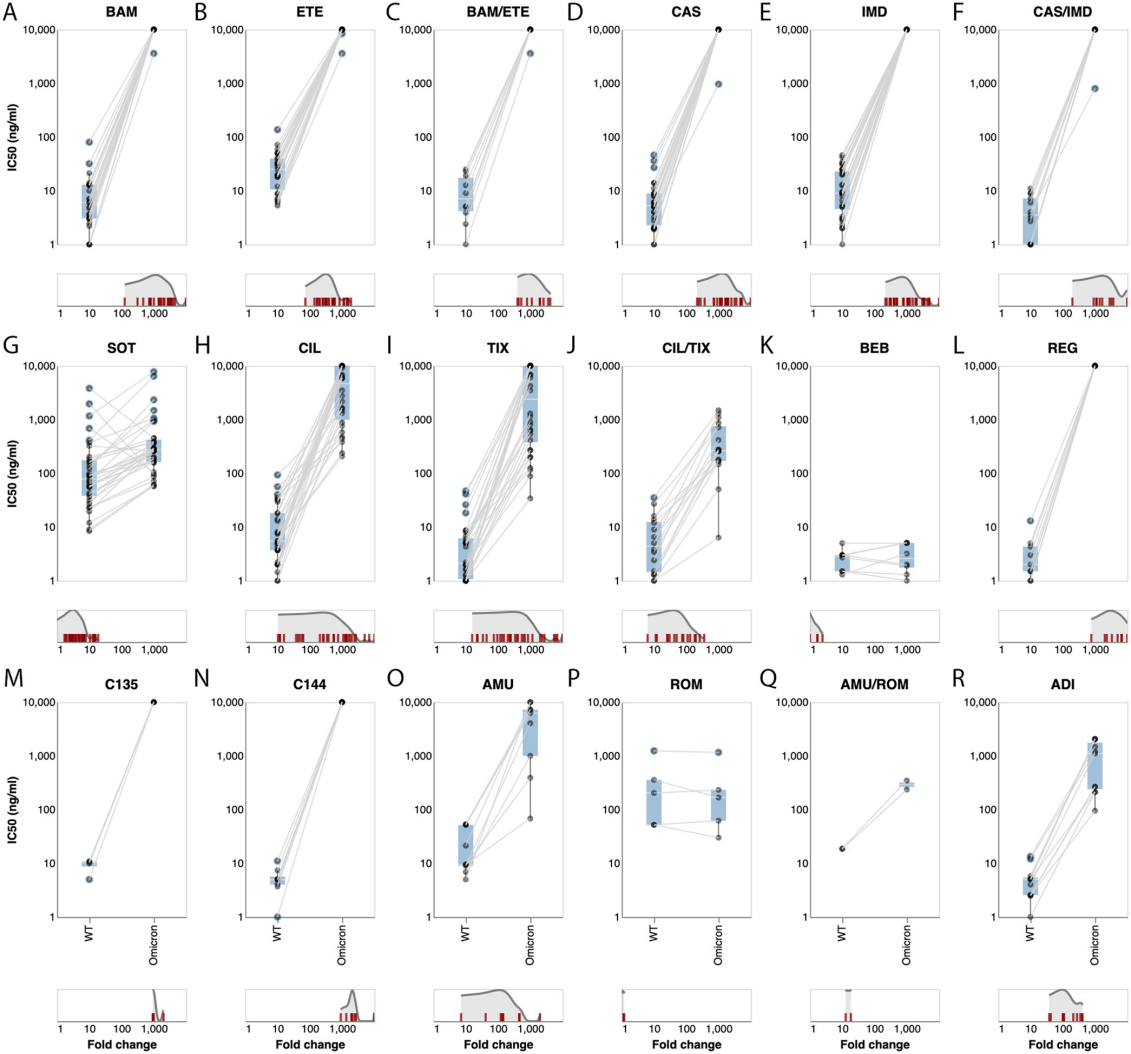
Neutralizing susceptibility to the Omicron BA.1 variant for 18 individual MAbs or MAb combinations. Each plot shows the IC_50_s of the wild-type control variant (on the left) connected by a line to the IC_50_s of the Omicron BA.1 variant (on the right) performed in the same study. The cyan boxes encompass the interquartile range. IC_50_s at or above 10,000 ng/mL or recorded as being above “>1,000 ng/mL” are plotted as 10,000 ng/mL. Several values below 1 ng/mL are plotted at 1 ng/mL. The distribution of fold reductions in susceptibility is shown beneath each plot. Studies that used a Delta variant control are not included in the plots.

Figures 3A to F display the 50% inhibitory concentrations (IC_50_s) against the Omicron BA.1 variant and a wild-type control for the MAbs in the first two FDA-authorized MAb preparations: bamlanivimab, etesevimab, and the combination bamlanivimab/etesevimab and casirivimab, imdevimab, and the combination casirivimab/imdevimab. In virtually all assays, the IC_50_ for each of these MAbs (alone and in combination) against Omicron was greater than 10,000 ng/mL and the reduction in their activity compared with control was approximately 1,000-fold.

### (ii) Sotrovimab.

Figure 3G displays the IC_50_s against the Omicron BA.1 variant and a wild-type control for sotrovimab (*n* = 37 results). For sotrovimab, the median wild-type IC_50_ was 78 ng/mL (interquartile range [IQR]: 38 to 176) and the median Omicron BA.1 variant IC_50_ was 276 ng/mL (IQR: 163 to 423). The median fold reduction in susceptibility (Omicron BA.1 IC_50_/wild-type IC_50_) was 4.0 (IQR: 2.6 to 6.9). There were four low (8.4 to 20 ng/mL) and seven high (324 to 3,819 ng/mL) wild-type outlier IC_50_s. There were four low (0.05- to 0.8-fold) and one high (19-fold) outlier for fold reduction in susceptibility.

One study using an authentic virus assay reported highly disparate results depending on whether Vero-TMPRSS2 cells (wild-type IC_50_ = 202 ng/mL and Omicron BA.1 variant IC_50_ = 373 ng/mL) or Vero-TMPRSS2-ACE2 cells (wild-type IC_50_ = 1,168 ng/mL and Omicron BA.1 variant IC_50_ = 7,756 ng/mL) were used (26). The four outlier studies with low fold reductions in susceptibility for the Omicron BA.1 variant used a lineage B control variant (lacking the D614G mutation) (15, 41, 42, 45).

### (iii) Cilgavimab/tixagevimab.

Figures 3H to J display the IC_50_s against the Omicron BA.1 variant and a wild-type control for cilgavimab (*n* = 32 results), tixagevimab (*n* = 34 results), and the combination cilgavimab/tixagevimab (*n* = 18 results). For cilgavimab, the median wild-type IC_50_ was 5.5 ng/mL (IQR: 3.7 to 18) and the median Omicron BA.1 variant IC_50_ was 4,669 ng/mL (IQR: 990 to 10,000). The median fold reduction in susceptibility was 553 (IQR: 62 to 1,628). There were two low (0.3 and 1 ng/mL) and seven high (30 to 93 ng/mL) wild-type outlier IC_50_s. There were 10 low (10- to 84-fold) and six high (2,425- to >10,000-fold) outliers for fold reduction in susceptibility.

For tixagevimab, the median wild-type IC_50_ was 2.1 ng/mL (IQR: 1.1 to 6.2) and the median Omicron BA.1 variant IC_50_ was 2,395 ng/mL (IQR: 379 to 10,000). The median fold reduction in susceptibility was 581 (IQR: 139 to 2,244). There were two low (0.1 and 0.2 ng/mL) and six high (8.6 to 47 ng/mL) wild-type outlier IC_50_s. There were nine low (16- to 135-fold) and nine high (2,326- to >10,000-fold) outliers for fold reduction in susceptibility.

For the combination cilgavimab/tixagevimab, the median wild-type IC_50_ was 4.4 ng/mL (IQR: 1.5 to 12) and the median Omicron BA.1 variant IC_50_ was 256 ng/mL (IQR: 170 to 750). The median fold reduction in susceptibility was 86 (IQR: 27 to 151). There were two low (0.1 and 0.5 ng/mL) and two high (27 and 35 ng/mL) wild-type outlier IC_50_s. There were three low (11- to 21-fold) and one high (359-fold) outlier for fold reduction in susceptibility.

### (iv) Bebtelovimab.

Figure 3K displays the IC_50_s against the Omicron BA.1 variant and a wild-type control for bebtelovimab (*n* = 11 results from 8 studies). For bebtelovimab, the median wild-type IC_50_ was 2.9 ng/mL (IQR: 1.5 to 3) and the median Omicron BA.1 variant IC_50_ was 2.6 ng/mL (IQR: 1.8 to 5.0). The median fold reduction in susceptibility was 1.0 (IQR: 0.7 to 1.4).

### (v) Non-FDA-authorized MAbs.

Regdanvimab, C135, C144, and amubarvimab displayed little residual activity against the Omicron BA.1 variant (Fig. 3L to O). Romlusevimab and adintrevimab (ADI) retained partial activity (Fig. 3P to R).

### Omicron BA.2 variant-neutralizing activity. (i) Bamlanivimab/etesevimab and casirivimab/imdevimab.

**FIG 4:**
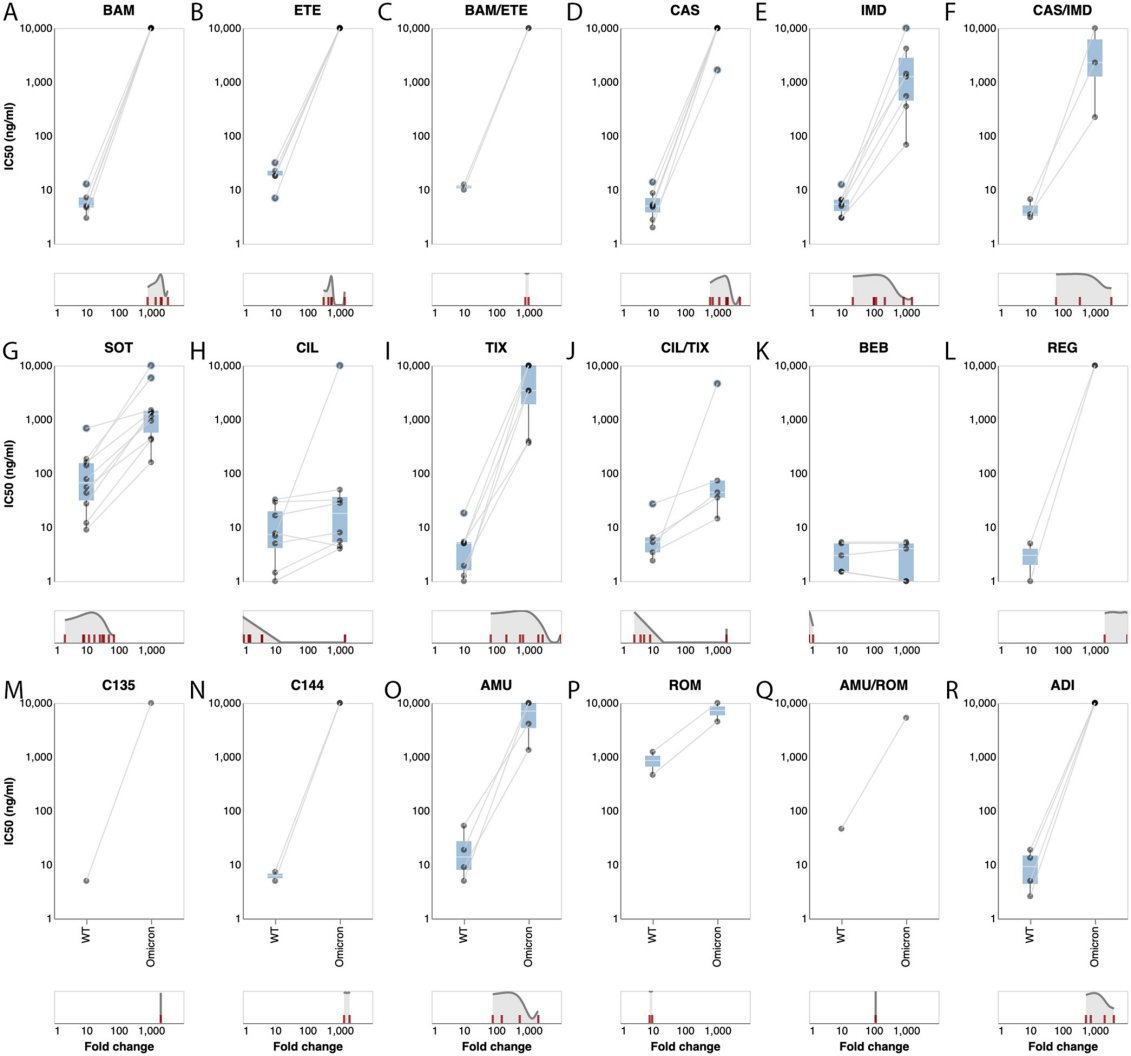
Neutralizing susceptibility to the Omicron BA.2 variant for 18 individual MAbs or MAb combinations. Each plot shows the IC_50_s of the wild-type control variant (on the left) connected by a line to the IC_50_s of the Omicron BA.1 variant (on the right) performed in the same study. The cyan boxes encompass the interquartile range. IC_50_s at or above 10,000 ng/mL or recorded as being above “>1,000 ng/mL” are plotted as 10,000 ng/mL. The distribution of fold reductions in susceptibility is shown beneath each plot. Two studies that used a Delta variant control are not included in the plots.

Figures 4A to C display the IC_50_s against the Omicron BA.2 variant and a wild-type control for bamlanivimab, etesevimab, and the combination bamlanivimab/etesevimab. In all assays, the IC_50_ for each of these MAbs (alone and in combination) against Omicron was greater than 10,000 ng/mL and the reduction in their activity compared with control was approximately 1,000-fold.

Figures 4D to F display the IC_50_s against the Omicron BA.2 variant and a wild-type control for casirivimab, imdevimab, and the combination casirivimab/imdevimab. For casirivimab, in virtually all assays, the IC_50_ against Omicron BA.2 was greater than 10,000 ng/mL and the reduction in activity compared with control was approximately 1,000-fold. For imdevimab, the median Omicron BA.2 variant IC_50_ was 1,253 ng/mL (IQR: 451 to 2,799) and the median fold reduction in susceptibility was 118 (IQR: 101 to 527). For casirivimab/imdevimab, the median Omicron BA.2 variant IC_50_ was 2,303 ng/mL (IQR: 1,263 to 6,152) and the median fold reduction in susceptibility was 344 (IQR: 203 to 1,785).

### (ii) Sotrovimab.

Figure 4G displays the IC_50_s against the Omicron BA.2 variant and a wild-type control for sotrovimab (*n* = 14 results). For sotrovimab, the median Omicron BA.2 variant IC_50_ was 1,250 ng/mL (IQR: 567 to 1,456). The median fold reduction in susceptibility was 17 (IQR: 13 to 30). There was one low (2.2-fold) and one high (71-fold) outlier for fold reduction in susceptibility.

### (iii) Cilgavimab/tixagevimab.

Figures 4H to J display the IC_50_s against the Omicron BA.2 variant and a wild-type control for cilgavimab (*n* = 9 results), tixagevimab (*n* = 8 results), and the combination cilgavimab/tixagevimab (*n* = 7 results). For cilgavimab, the median Omicron BA.2 variant IC_50_ was 18 ng/mL (IQR: 5.3 to 36) and the median fold reduction in susceptibility was 1.7 (IQR: 1.5 to 3.9). There was one high (1,449-fold) outlier for fold reduction in susceptibility.

For tixagevimab, the median Omicron BA.2 variant IC_50_ was 3,438 ng/mL (IQR: 1,908 to 10,000) and the median fold reduction in susceptibility was 837 (IQR: 461 to 2,179). There were two low (68- and 206-fold) and one high (>10,000-fold) outlier for fold reduction in susceptibility.

For the combination cilgavimab/tixagevimab, the median Omicron BA.2 variant IC_50_ was 44 ng/mL (IQR: 35 to 73) and the median fold reduction in susceptibility was 5.4 (IQR: 3.7 to 6.9). There was one high (1,920-fold) outlier for fold reduction in susceptibility.

### (iv) Bebtelovimab.

Figure 4K displays the IC_50_s against the Omicron BA.2 variant. The median Omicron BA.2 variant IC_50_ was 4.0 ng/mL (IQR: 0.8 to 5.0) and the median fold reduction in susceptibility was 1.0 (IQR: 0.7 to 1).

### (v) Non-FDA-authorized MAbs.

Regdanvimab, C135, C144, romlusevimab, ADI, and amubarvimab displayed little residual activity against the Omicron BA.2 variant (Fig. 4J, L to R).

### Omicron BA.1.1 (BA.1 ± R346K) neutralizing susceptibility data.

The susceptibility of pseudotyped viruses containing the prototypical Omicron BA.1 variant mutations plus R346K (also referred to as Omicron BA.1.1) was evaluated in 11 studies. In these studies, the addition of R346K was found to reduce cilgavimab activity by 5- to 10-fold (13, 19) and romlusevimab activity by more than 10-fold (19) compared with Omicron BA.1 lacking R346K but to have no impact on sotrovimab susceptibility (12, 17, 34, 36, 41, 55, 57).

### Effects of the individual mutations in Omicron BA.1 and BA.2 on MAb susceptibility.

Six studies evaluated the susceptibility of the individual RBD mutations present in both BA.1 and BA.2 to between 5 and 11 MAbs (17, 19, 29, 32, 34, 54). Ten of the RBD mutations reduced susceptibility to one or more authorized MAbs by a median of ≥5-fold: (i) S371F reduced susceptibility to etesevimab (143- to 630-fold), casirivimab (14- to 28-fold), imdevimab (11- to 126-fold), sotrovimab (5.5- to 21-fold), and tixagevimab (6.3- to 31-fold); (ii) S371L reduced susceptibility to etesevimab (6.2- to 31-fold), imdevimab (11- to 74-fold), and sotrovimab (7.4- to 240-fold); (iii) D405N reduced susceptibility to etesevimab (16- to 26-fold) and casirivimab (11- to 14-fold); (iv) K417N reduced susceptibility to etesevimab (>100-fold) and casirivimab (6.4- to 249-fold); (v) N440K reduced susceptibility to imdevimab (9.9- to 246-fold); (vi) G446S reduced susceptibility to imdevimab (>100-fold) and cilgavimab (8- to 12-fold); (vii) E484A reduced susceptibility to bamlanivimab (>100-fold), casirivimab (9- to 22-fold), and tixagevimab (8- to 11-fold); (viii) Q493R reduced susceptibility to bamlanivimab (>100-fold), etesevimab (16- to 65-fold), and casirivimab (42- to 56-fold); (ix) G496S reduced susceptibility to imdevimab (6.2- to 8.2-fold); and (x) N501Y reduced susceptibility to etesevimab (9.8- to 23-fold). Table S3 in the supplemental material lists the IC_50_s and fold reductions in susceptibility for each individual RBD mutation by reference and MAb.

### Effect of method on neutralizing susceptibility.

**FIG 5:**
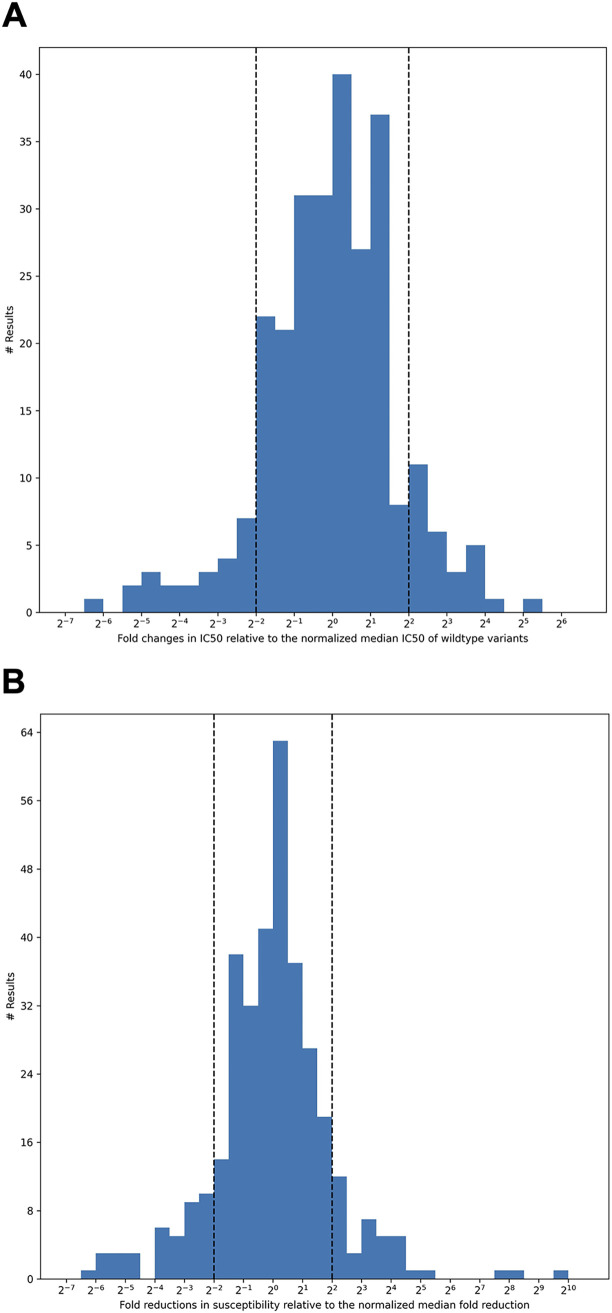
The distribution of fold changes in IC_50_s relative to the normalized median IC_50_ for all authorized MAbs against wild-type variants (A) and the distribution of fold reductions in susceptibility (Omicron variant IC_50_/wild-type control IC_50_) relative to the normalized median fold reduction for all authorized MAbs (B). Results that were more than 4-fold (2^−2^) below or 4-fold (2^2^) above the median result for an MAb were classified as outliers.

Disparities among assays both for the wild-type variants and for Omicron BA.1 and BA.2 were evident by the wide spread in results for the same MAb in different assays. Figure 5A shows the distribution in the IC_50_s for wild-type variants normalized to the median for each MAb. For all 268 results, 129 (48.1%) were between 2-fold lower and 2-fold higher than the normalized median wild-type IC_50_, 217 (81.0%) were between 4-fold lower and 4-fold higher than the normalized median, and 51 (19.0%) were outliers.

Figure 5B shows the spread in the fold reduction in susceptibility for the pooled Omicron BA.1 and BA.2 variants normalized to the median for all MAbs. For all 348 results, 173 (49.7%) were between 2-fold lower and 2-fold higher than the normalized median fold reduction in susceptibility, 270 (77.6%) were between 4-fold lower and 4-fold higher than the normalized median fold, and 78 (22.4%) were outliers.

**FIG 6:**
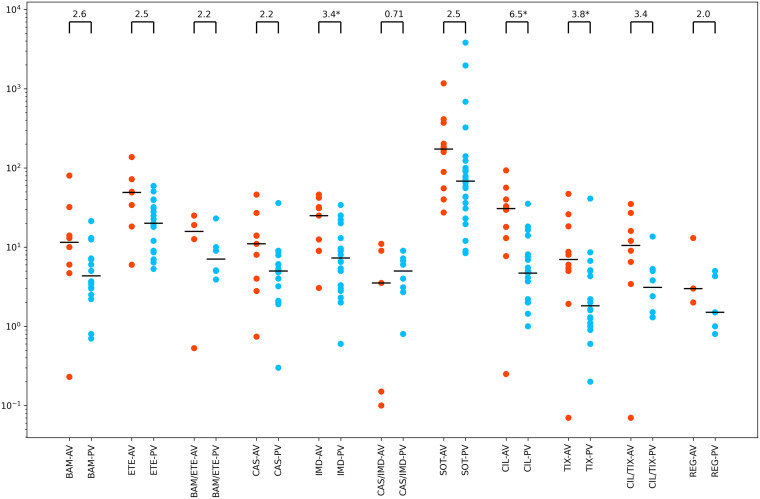
Neutralizing susceptibility for MAbs for which two or more infectious virus (authentic virus [AV]) assays (red points) and two or more pseudotyped virus (PV) assays (blue points) were performed. Such results were available for each of the authorized MAbs (both individually and in combination) except for bebtelovimab, for which just PV assays were available. Results were also available for regdanvimab (REG). Horizontal lines indicate median values. Fold changes are indicated at the top of each plot. Those with asterisks have a *P* value of <0.05 by the Wilcoxon rank sum test. BAM, bamlanivimab; ETE, etesevimab; CAS, casirivimab; IMD, imdevimab; SOT, sotrovimab; CIL, cilgavimab; TIX, tixagevimab.

To determine whether the use of authentic viruses as opposed to pseudotyped viruses influenced susceptibility, we compared the median IC_50_s for 11 individual and combination MAbs against wild-type virus for those MAbs undergoing at least two authentic virus assays (median 8 assays) and at least two pseudotyped virus assays (median 21 assays). This analysis showed that the median of the authentic virus assays was greater than the median of the pseudotyped virus assays for 10 of the 11 individual and combination MAbs (Fig. 6). The median ratio of the fold difference between the authentic virus median and the pseudotyped virus assay was 2.6 (range: 0.7 to 6.5).

It was not possible to determine whether the cell line influenced MAb IC_50_s against wild-type viruses because nearly all authentic virus assays used Vero cells while no difference between 293T and Vero cells was observed in the pseudotyped virus assays. Although one study showed that the sotrovimab IC_50_ for both wild-type virus and Omicron was higher when Vero-ACE2-TMPRSS2 cells were used than when Vero-TMPRSS2 cells were employed, we were unable to demonstrate whether cells that overexpressed ACE2 or TMPRSS2 consistently had different IC_50_s than other cell lines.

## DISCUSSION

In this systematic review, Omicron BA.1 and BA.2 were highly resistant to the first two authorized MAb combinations, bamlanivimab/etesevimab and casirivimab/imdevimab. In virtually all cases, the IC_50_s for these MAbs were above the upper limits of quantification. The third and fourth authorized MAb preparations, sotrovimab and cilgavimab/tixagevimab, displayed much lower reductions in susceptibility to these variants. However, there was a high degree of disparity among studies reporting the susceptibility of Omicron BA.1 and BA.2 to these MAb preparations. The fifth authorized MAb, bebtelovimab, retained *in vitro* activity against Omicron BA.1 and BA.2.

The median fold reductions in Omicron BA.1 and BA.2 susceptibility to sotrovimab were 4.0-fold (IQR: 2.6 to 6.9) and 17-fold (IQR: 13 to 30), respectively. The median fold reductions in Omicron BA.1 and BA.2 susceptibility to cilgavimab/tixagevimab were 86-fold (IQR: 27 to 151) and 5.4-fold (IQR: 3.7 to 6.9), respectively. The increased susceptibility of Omicron BA.2 relative to Omicron BA.1 for cilgavimab/tixagevimab resulted from the nearly complete restoration in susceptibility of BA.2 to cilgavimab.

As a result of the reduced susceptibility of cilgavimab/tixagevimab to the BA.1 variant, the FDA recommended on 24 February 2022 that the dosage for each MAb in this combination be increased from 150 mg to 300 mg intramuscularly. As a result of the high proportion of cases in the United States arising from Omicron BA.2, the FDA discontinued the authorization of sotrovimab for treating SARS-CoV-2 infections on 5 April 2022. Despite limited clinical efficacy data, bebtelovimab was authorized for the outpatient treatment of high-risk patients with COVID-19 primarily based on its *in vitro* activity (56). As of this writing, there have been no structural studies that explain precisely how the binding mode of bebtelovimab avoids Omicron BA.1 and BA.2 mutations or how the reintroduction of glycines at positions 446 and 496 in Omicron BA.2 restores cilgavimab neutralizing activity.

Neutralization assays can be characterized by several variables including the nature of the virus preparation, the cell line used, the size of the virus inoculum, and the duration and means of quantifying virus entry into cells (60, 61). The two main types of virus preparation employed in the studies reviewed here were pseudotyped and authentic virus assays. A previous meta-analysis of the neutralizing susceptibility of Omicron BA.1 to the polyclonal Abs in the plasma from convalescent and/or vaccinated individuals reported that pseudotyped virus assays yielded two- to three-times-higher geometric mean titers than did authentic virus assays for certain subpopulations (62). Our comparison of the IC_50_s of the most common MAb preparations against wild-type variants supports this observation, showing a median 2.6-fold-increased IC_50_ for assays performed using authentic viruses.

The nature of the virus preparation influences several additional aspects of neutralization assays. Authentic virus assays are influenced by differences in virus mutations outside the spike gene and potentially by mutations that arise during the process of virus isolation. Although complete genomic sequence data were not available for all of the virus isolates studied, the reported spike sequences were nearly identical in all assays with minor differences reported in several studies. Authentic virus assays may also be more likely to be affected by the duration of virus culture if cell-to-cell spread is not restricted, whereas pseudotyped viruses replicate only in the first cell they enter. Finally, authentic virus assays measure cytopathic effect usually augmented by immunostaining of virally infected cells while pseudotyped virus assays measure relative light units produced by luciferase-encoding reporter genes.

The most commonly used cell lines were African green monkey kidney epithelial Vero cells and human embryonic kidney (HEK) 293T cells. As Vero cells are resistant to HIV-1 infection, 293T cells were used for all pseudotyped HIV-1 assays. Depending on the study, both types of cells were modified to overexpress ACE2, TMPRSS2, or both receptors (Table 1). Although it was not possible to discern an overall effect of cell line on IC_50_ values, one study showed that the sotrovimab IC_50_ against both wild-type and Omicron viruses was higher for Vero-ACE2-TMPRSS2 cells than for Vero-TMPRSS2 cells (26), which is consistent with an observation from another study which reported that the IC_50_ of sotrovimab against wild-type isolates was lowest for Vero and Huh-7 cells and highest for cells overexpressing ACE2 (63). That study also proposed that part of the activity of sotrovimab may be related to its inhibition of the interaction of spike and transmembrane lectins, which function as attachment inhibitors (63).

With the exception of four studies, the choice of the wild-type control variant did not appear to impact MAb susceptibilities (15, 41, 42, 45). In these studies, a wild-type lineage B virus (lacking D614G) was less susceptible to sotrovimab than the Omicron BA.1 variant (which contains D614G). One previous study has also reported that sotrovimab was 162 times more active against pseudotyped viruses containing D614G than against those lacking this mutation (64).

The size of the virus inoculum influences the IC_50_ for an antiviral agent by shifting the dose-response curve. However, the virus inoculum size was not consistently reported for either the authentic or the pseudotyped virus assays. Moreover, when it was reported, different measurements were employed. Authentic virus assays reported the virus inoculum size as either a 50% tissue culture infectious dose (TCID_50_), the number of focus-forming units, or a multiplicity of infection. Pseudotyped virus assays reported the inoculum size as a TCID_50_ or as the number of relative light units. Establishing a relationship between virus inoculum and IC_50_ was further complicated because the use of a highly permissive cell line may have the same effect as using a larger virus inoculum.

Each of the authorized MAbs has been reported to be highly potent *in vitro* against wild-type SARS-CoV-2 variants and to reduce virus loads and the severity of infection in one or more animal models. Each of the authorized MAbs except for bebtelovimab has been shown in phase III clinical trials to prevent infection and/or reduce the risk of disease progression in high-risk outpatients infected with ancestral wild-type variants. There is a long tradition of considering findings about the level of *in vitro* antiviral resistance to be clinically significant and informative. With the emergence of SARS-CoV-2 variants containing spike mutations conferring reduced MAb susceptibility, it has become important to quantify the reduction in susceptibility to ascertain whether a MAb is likely to be as effective as previously reported for earlier variants, to ascertain whether an increase in dosage may be required, and to prioritize its use over other antiviral treatment options.

In the treatment of viruses with reduced susceptibility, pharmacokinetics attains greater clinical significance because if the obtainable antiviral levels in patients are insufficient, viral suppression may not be possible. For sotrovimab, the geometric mean maximum concentration of drug in serum (*C*_max_) in approximately 300 patients following a 1-h 500-mg intravenous infusion was 117.6 μg/mL with concentrations decreasing to 24.5 μg/mL after 1 month (55). For cilgavimab/tixagevimab, the *C*_max_ following an intramuscular injection of 150 mg of each MAb was 16.5 and 15.3 μg/mL, respectively, with levels between 4 and 6 μg/mL being present for as long as 6 months (57). Thus, the very high concentrations achievable with these SARS-CoV-2 MAbs, well above the median Omicron IC_50_s, may mitigate their reduced activity. For most antiviral agents, combination therapy has been beneficial because each agent prevents the emergence of resistance to the other agent. However, the *in vitro* synergy between cilgavimab and tixagevimab may reflect the fact that the two MAbs can simultaneously bind to the spike RBD at nonoverlapping epitopes (65). How often the two MAbs bind simultaneously to the trimeric spike *in vivo*, however, is likely to be difficult to determine.

Some limitations of our review should be discussed. We included data from peer-reviewed papers, preprints, and one database, and thus, some included information is not fully peer reviewed. Moreover, it is possible that additional studies with relevant results have remained unavailable to date, but there is no strong reason to suspect that their findings would be systematically different from those that were available for this review. Moreover, in a rapidly emerging field, early results may have more heterogeneity than when the full picture emerges. Finally, some study characteristics were not sufficient in full detail.

In conclusion, the marked variability in results reported in different studies is concerning and complicates the interpretation of published findings. Because many methodological aspects can influence neutralizing susceptibility, we were unable to determine the reason for the disparities between assays for the activity of the same MAb or MAb combination against wild-type viruses and the Omicron variants. One approach would be to standardize the method for assessing neutralizing activity (61). Another approach would be to use an external standard, such as that provided by the World Health Organization (WHO), to calibrate the results of assays performed under different conditions (61, 66). However, this reagent’s limited and nonrenewable nature currently limits its wide-scale utilization (67). The marked loss of activity of many MAbs against the Omicron variants also underscores the importance of developing MAbs that target conserved regions of spike that are not targeted by the antibodies produced in infected persons.

## MATERIALS AND METHODS

We searched PubMed, bioRxiv, medRxiv, and Research Square using the search terms “SARS-CoV-2 AND Omicron AND (Antibody OR Neutralization OR Therapy)” to identify studies in which SARS-CoV-2 Omicron variants were assessed for their neutralizing susceptibility to FDA-authorized MAbs. We supplemented the data in these studies with data from the FDA fact sheets for sotrovimab, cilgavimab/tixagevimab, and bebtelovimab and with data available on an NIH website provided by two MAb manufacturers (https://opendata.ncats.nih.gov/covid19/). This is a living systematic review with the plan to update results as more studies become available. For the analyses presented here, searches were last updated on 11 April 2022.

Each study was reviewed to determine the IC_50_ in nanograms per milliliter of MAbs against the Omicron BA.1, BA.1.1, and BA.2 variants and against control SARS-CoV-2 variants defined as those lacking RBD mutations. In addition to susceptibility data for authorized MAbs (bamlanivimab, etesevimab, casirivimab, imdevimab, sotrovimab, cilgavimab, tixagevimab, and bebtelovimab), we also collected susceptibility data for six additional MAbs including three that have been approved in another country (regdanvimab, amubarvimab, and romlusevimab) and three that are in phase II or III clinical trials (adintrevimab, C135, and C144). For each study, the sequence of the wild-type variant and the RBD mutations in the Omicron variant were recorded. Mutations were defined as amino acid differences from the Wuhan-Hu-1 reference variant. Most control variants matched the Wuhan-Hu-1 variant or belonged to an early clade A or B variant (including B.1 variants which contain the spike mutation D614G, which emerged early in the pandemic) or other variants lacking RBD mutations. For several studies, a Delta variant served as the control.

For each study, we recorded the following information about the assays employed to assess neutralizing activity: (i) whether the virus used to assess neutralization was an infectious virus isolate (also referred to as an “authentic virus”) or a non-replication-competent pseudotyped virus, (ii) the cell line used to assess neutralization, (iii) the virus inoculum size, (iv) the duration of the assay, (v) the method used to assess either virus replication for authentic virus assays or cell entry for pseudotyped virus assays, and (vi) the highest MAb concentration employed and whether dose-response curves were included in the study’s publication. Although some studies referred to the tested MAbs by their generic names and others by the name of their parent compounds, it was rarely known whether these MAbs differed from one another. Therefore, we referred to all such MAbs by their generic name.

All IC_50_s were reported in nanograms per milliliter. For the purposes of analysis, IC_50_s reported as being “>1,000 ng/mL” or as being above 10,000 ng/mL were both recorded as 10,000 ng/mL. IC_50_s reported as being between 1,000 ng/mL and 10,000 ng/mL were recorded as the value provided by the study. Fold reductions in susceptibility were not analyzed for four studies that used a Delta virus variant rather than a wild-type control virus variant. Outliers were defined as values that were more than 4-fold lower or 4-fold higher than the overall median of all assay results for the same MAb. Outliers were included in calculating the median.

Supplemental File 1 lists each of the individual IC_50_s for Omicron BA.1, Omicron BA.2, Omicron BA.1.1, and the control variant and the fold reductions in susceptibility (i.e., ratio of the Omicron variant/control variant). Supplemental File 2 contains the median, IQR, and range for each result along with the list of outliers for each variant and MAb. Supplemental File 3 lists the IC_50_s and fold reductions in susceptibility for each of the individual Omicron spike RBD mutations.

Screening for eligibility and data extraction were performed independently by two of the authors. Given the large between-study heterogeneity in several of the estimates, we focused on median and interquartile range whenever appropriate rather than a formal quantitative synthesis with meta-analysis. However, as we were particularly interested in disparities between assays, we also reported the full range in susceptibility values between different assays for the same MAb. Each of the assay characteristics was examined as a source that might explain the heterogeneity between subgroups of studies, but no formal quantitative synthesis of subgroup data was performed because of the small number of studies sharing the same assay characteristics. The reporting of the overview follows the PRISMA guidelines (68).

### Data availability.

The data for this project can be found on the GitHub repository https://github.com/hivdb/covid-drdb-payload. The code can be found on the GitHub repository https://github.com/hivdb/covid-drdb. The code for Fig. 3 and 4 can be found at https://observablehq.com/@2a230210780ca54d/mab-neutralization-review.
